# Effect of Exogenous Abscisic Acid and Methyl Jasmonate on Anthocyanin Composition, Fatty Acids, and Volatile Compounds of Cabernet Sauvignon (*Vitis vinifera* L.) Grape Berries

**DOI:** 10.3390/molecules21101354

**Published:** 2016-10-12

**Authors:** Yan-Lun Ju, Min Liu, Hui Zhao, Jiang-Fei Meng, Yu-Lin Fang

**Affiliations:** 1 College of Enology, Northwest A&F University, Yangling 712100, Shaanxi, China; juyanlun@163.com (Y.-L.J.); liumin272@163.com (M.L.); zhaohuizh163@163.com (H.Z.); mjfwine@nwsuaf.edu.cn (J.-F.M.); 2 Shaanxi Engineering Research Center for Viti-Viniculture, Yangling 712100, Shaanxi, China

**Keywords:** plant hormones, physicochemical, volatile aroma, Cabernet Sauvignon

## Abstract

The anthocyanin composition, fatty acids, and volatile aromas are important for Cabernet Sauvignon grape quality. This study evaluated the effect of exogenous abscisic acid (ABA) and methyl jasmonate (MeJA) on the anthocyanin composition, fatty acids, lipoxygenase activity, and the volatile compounds of Cabernet Sauvignon grape berries. Exogenous ABA and MeJA improved the content of total anthocyanins (TAC) and individual anthocyanins. Lipoxygenase (LOX) activity also increased after treatment. Furthermore, 16 fatty acids were detected. The linoleic acid concentration gradually increased with ABA concentration. The fatty acid content decreased with increasing MeJA concentration and then increased again, with the exception of linoleic acid. After exogenous ABA and MeJA treatment, the C6 aroma content increased significantly. Interestingly, the exogenous ABA and MeJA treatments improved mainly the content of 1-hexanol, hexanal, and 2-heptanol. These results provide insight into the effect of plant hormones on wine grapes, which is useful for grape quality improvement.

## 1. Introduction

Grapes are one of the most important fruits because of their low cost and nutritional benefits, and are widely cultivated around the world. The physicochemical composition of grapes is a key factor in determining the taste and color of the berries. As it is widely known, the content of anthocyanins in grape berries at harvest is one of the most important indicators to measure the quality of the fruit [1]. Especially for wine grapes, the content of anthocyanins in berries plays an important role in determining their quality [2,3]. Aroma is one of the most important characteristics for grape berry quality, for which C6 and C9 volatiles are important aroma compounds mainly formed in the lipoxygenase (LOX; *EC 1.13.11.12*) pathway, and are responsible for the “green” fresh notes in wine [4,5].

LOX is an important enzyme in the formation of green leaf volatile substances. Furthermore, LOX is ubiquitous in plants, being present in leaves, stems, roots, flowers, fruit pulp, and seeds [6,7]. In addition, LOX can catalyze the oxidation of polyunsaturated lipids and esters that contain a 1,4-*cis*,*cis*-pentadiene structure, such as linoleic and linolenic acids, into conjugated hydroperoxides (HPOs) [8]. HPOs are produced by different reactions with different enzymes, and the products are directly involved in the formation of the fruit aroma, which determines the fruit quality [9]. Meanwhile, LOX can regulate the production of jasmonic acid and improve plant resistance. In addition, the LOX activity affects the flavor quality during food storage [10].

Fatty acids are substrates formed from volatiles and are the main precursor for the formation of grape flavor [11]. LOX can also catalyze the transformation of fatty acids to hydroperoxides, which can be catalyzed by hydroperoxide lyase (HPL) to form small volatile molecules, such as alcohols, aldehydes, and esters [12]. These substances are important sources of grape berry, grape juice, and wine aroma [13].

Abscisic acid (ABA) has a positive regulatory effect on plant growth, and it protects plants from environmental stress. Treatment with ABA can provoke a variety of metabolic problems in plants and can affect various aspects of plants [14]. According to a previous study, ABA can regulate the expression of structure synthesis genes and regulate genes in the anthocyanin pathway [2,3]; therefore, ABA plays an important role in the promotion of fruit ripening and fruit anthocyanin content [15]. In addition, methyl jasmonate (MeJA) regulates the LOX activity involved in the fatty acid metabolic pathway [16], and so affects the synthesis of small molecules. Overall, it is important to study how both hormones influence the plant growth and fruit quality.

In recent years, most research has focused on the effect of environmental conditions, exogenous hormones, or management practices on grape berry volatile compounds [17,18,19], and just a few studies have reported the effect of exogenous ABA and MeJA on the physicochemical composition and volatile compounds formed in the LOX pathway of grape berries, especially those regarding C6 and C9 volatiles. In this work, we have investigated the development of chemical species and volatile compounds derived from fatty acids through the LOX pathway in Cabernet Sauvignon grapes. The study focuses in particular on the relationship between fatty acids, enzyme activity, and volatile production after exogenous ABA and MeJA treatments. These results are useful for understanding the accumulation of C6 and C9 compounds derived from fatty acids in grape berries.

## 2. Results and Discussion

### 2.1. Physico-Chemical Properties and TPC and TAC Analysis

The quality of grape berries is affected by rainfall, light, temperature, and other environmental factors. At the same time, other cultivation practices, such as exogenous hormone treatments, also directly affect the fruit quality [20,21]. As a result, after application of exogenous ABA, the Brix of grape berries increases. As previously reported, ABA is involved in the transformation of sugar and organic acids in fruit [22,23], which explains why exogenous ABA can increase the sugar content of ripe berries. Exogenous MeJA has no effect on the Brix of grape berries. As it is widely known, polyphenols and anthocyanins are essential for the color and taste of grapes [2,3]. Exogenous ABA and MeJA increase the total content of polyphenols and anthocyanins (Table 1). In particular, after ABA treatment, the content of anthocyanins increased to 12.28 mg·g^−1^, significantly higher than in the control sample (8.85 mg·g^−1^). Exogenous MeJA treatment increased the content of polyphenols to 22.19 mg·g^−1^. This might be due to the enhanced expression of key genes (*PAL*, *CHI* and *MYB*) in the synthesis pathway of polyphenols and anthocyanins after treatment with ABA and MeJA, which thus promote the synthesis of polyphenols and anthocyanins [23].

### 2.2. Effect of Exogenous ABA and MeJA on Individual Anthocyanins

**Figure 1:**
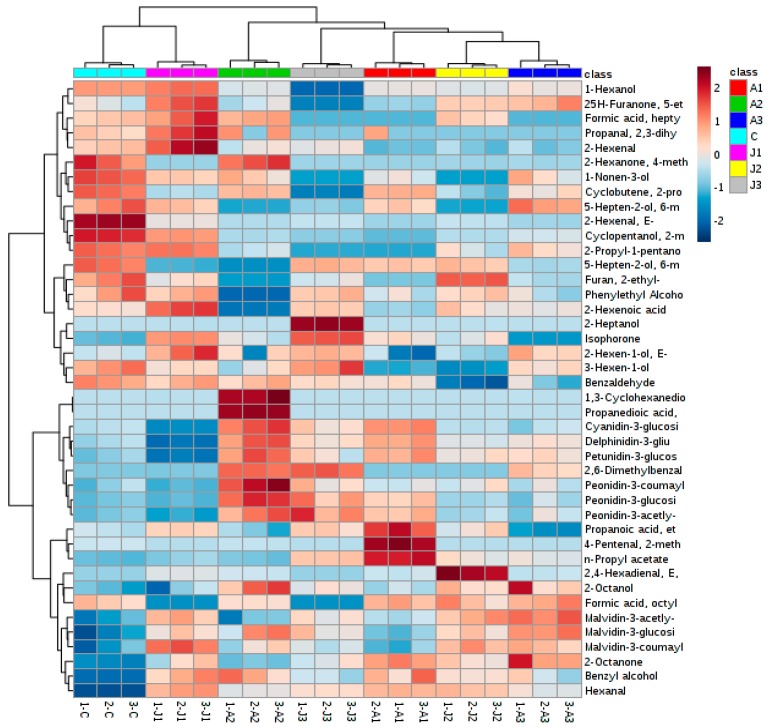
Heat map of anthocyanins and volatile aromas after different treatments. A1: treatment with 1000 mg·L^−1^ ABA; A2: treatment with 600 mg·L^−1^ ABA; A3: treatment with 200 mg·L^−1^ ABA; J1: treatment with 800 µmol·L^−1^ MeJA; J2: treatment with 200 µmol·L^−1^ MeJA; J3: treatment with 50 µmol·L^−1^ MeJA; C: control. Values are the mean of three replicates (±standard deviation). The heat map graphic distances were measured using Euclidean distances, and the clustering algorithm using ward dendrogram. Each colored cell on the map corresponds to a concentration value (normalized to the control sample).

In grape berries, the glucose-6 moiety of anthocyanins can undergo acetylation modification reactions, such as flower pigment acetylation, coffee acetylation, coumaric acid of glucoside. These modification reactions affect the color and stability of anthocyanins [21]. It has been reported that exogenous ABA and MeJA treatments promote the synthesis of anthocyanins [2]. Our study shows that exogenous ABA and MeJA treatment can improve the total anthocyanin content, and has a significant effect on the content of individual anthocyanins in grape berries (Table 1 and Table 2). The effect of exogenous ABA and MeJA treatments on various monomeric anthocyanins is presented in Table 2 and Figure 1, in comparison with the results for the control sample. The content of non-acetylated anthocyanins increased with the treatment concentration (1000 mg·L^−1^ and 600 mg·L^−1^), but the content of acetylation and coumaric acylated anthocyanins decreased slightly. In contrast, both acetylation and coumaric acylated anthocyanins increased at low concentration treatment (200 mg·L^−1^), similarly to the results reported by Luan [21]. MeJA treatment had little effect on the content of non-acetylated anthocyanins, but enhanced the content of acetylated and coumaric acylated anthocyanins. Treatment with ABA and MeJA mainly affected the content of non-acetylated anthocyanins, especially the ABA treatment, which significantly affected the content of malvidin-3-*O*-glucoside, cyanidin-3-*O*-glucoside, and delphinidin-3-*O*-glucoside (Figure 1). The treatment improved the expression of structure synthesis genes and the regulation of genes in the anthocyanin pathway, so that the content of anthocyanins such as petunidin, delphinidin, cyanidin, and peonidin increased in the skin. Thus, the stability of the pigment increased, directly affecting the quality of the wine [3,22]. In this study, the content of non-acetylated anthocyanins were most (about 70%), followed by acetylation of anthocyanins in grape berries (Table 2). These results are consistent with those by Núñez et al. [23], who used Cabernet Sauvignon, Tempranillo, and Graciano grapes as source materials. These results demonstrate that although different types of anthocyanins in different grape cultivars are acetylated in different ways, non-acetylated and acetylated anthocyanins are the major anthocyanin components in grape berries. As shown in Figure 1, treatment J2 is clustered with treatment A3, which might be due to the two treatments affecting the content of malvidin and petunidin in a similar way. Treatment J1 is clustered with treatment C, possibly due to the two treatments affecting mainly the volatile content. The A2 treatment is found next to J treatments, since the A2 treatment changes the content of propyl-propanedioic acid, *cis*-1,3-cyclohexanediol, petunidin, delphinidin, cyanidin, and peonidin.

### 2.3. Effect of Exogenous ABA and MeJA on Fatty Acid Composition and LOX Activity

#### 2.3.1. Analysis of Fatty Acid Composition

Fatty acids are the main precursors for the formation of fruit flavors [24]. LOX mainly catalyzes the conversion of linoleic acid or linolenic acid into conjugated hydroperoxides (HPOs). HPOs are produced by different reactions with different enzymes; the products are directly involved in the formation of fruit aromas (C6 or C9 aldehydes and alcohols) [25,26]. As shown in Table S1, 16 fatty acids were detected in Cabernet Sauvignon grapes. The polyunsaturated fatty acid (PUFA) content consisted mainly of linoleic acid, oleic acid, and palmitoleic acid; however, no linolenic acid was detected. In addition, the monounsaturated fatty acid (MUFA) content consisted primarily of palmitic acid, stearic acid, behenic acid, and arachidic acid. Table 3 shows large concentrations of PUFA (207.93 and 281.98 mg·kg^−1^) including linoleic acid (LA, 18:2n-6), from 116.75 to 178.08 mg·kg^−1^, in all groups, and concentrations of MUFA from 152.58 to 240.45 mg·kg^−1^. PUFA content was found to be higher than that of MUFA in all samples, in agreement with previously reported data [27,28]. The linoleic acid concentration gradually increased with the ABA concentration. The linoleic acid content was the lowest when the ABA concentration reached 1000 mg·L^−1^ and was the highest when the ABA concentration was 600 mg·L^−1^. The other fatty acid concentrations increased after ABA treatment. As the MeJA concentration increased, the fatty acid content initially decreased and then increased, with the exception of linoleic acid. In terms of linoleic acid, the content was lower than that in the control sample (Table 3). This may be ascribed to the exogenous hormone treatment promoting the direct reaction of linoleic acid as a substrate of LOX, converting linoleic acid into small molecules, such as alcohols, aldehydes, and esters [5,29].

#### 2.3.2. Determination of LOX Activity

**Figure 2:**
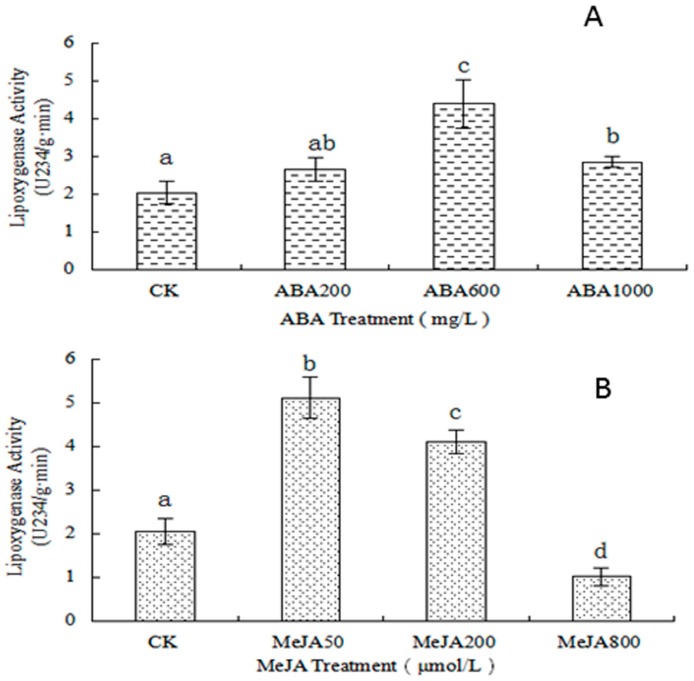
Lipoxygenase activity of Cabernet Sauvignon berries after different treatments. (**A**) ABA treatment; (**B**) MeJA treatment. Experiments were repeated three times. Data are the mean ± SD of replicates. ABA1000: treatment with 1000 mg·L^−1^ ABA; ABA600: treatment with 600 mg·L^−1^ ABA; ABA200: treatment with 200 mg·L^−1^ ABA; MeJA800: treatment with 800 µmol·L^−1^ MeJA; MeJA200: treatment with 200 µmol·L^−1^ MeJA; MeJA50: treatment with 50 µmol·L^−1^ MeJA; C: control. Different letters within a column indicate statistically significant differences between the means (*p* < 0.05).

LOX exerts an essential role in the fatty acid metabolic pathway, converting PUFA (mainly linoleic and linolenic acids) to HPOs and, after a series of reactions, generating aldehydes or jasmonate (JA) [7,11,30]. The LOX activity increased with the ABA concentration, except at 1000 mg·L^−1^ ABA (Figure 2A). The LOX activity was the highest when the ABA concentration was 600 mg·L^−1^. The LOX activity of the treated samples was significantly higher than that of the control sample. As shown in Figure 2B, the LOX activity was the highest when the MeJA concentration was 50 μmol·L^−1^ (more than 2.5 fold that of the control group) and was the lowest when the MeJA concentration was 800 μmol·L^−1^. This can be explained by exogenous ABA and MeJA improving the LOX activity by catalyzing more linoleic acid into small molecules [28] and more C6 and C9 aromatic products (Table 3 and Table S2). This may in turn be caused by enhanced LOX gene expression, increasing the LOX activity [4].

### 2.4. Effect of Exogenous ABA and MeJA on Volatile Aromas

**Figure 3:**
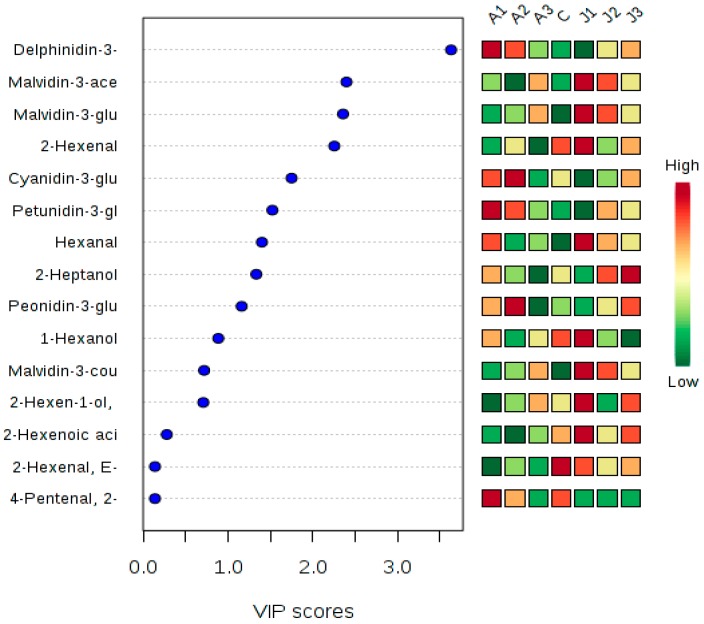
Important features identified by PLS-DA. Partial Least Squares-Discriminant Analysis (PLS-DA) model was built between the data (X) and the permuted class labels (Y) using the optimal number of components determined by cross validation for the model based on the original class assignment. Variable Importance in Projection (VIP) is a weighted sum of squares of the PLS loadings taking into account the amount of explained Y-variation in each dimension. VIP scores are calculated for each component. The colored boxes on the right indicate the relative concentration of the corresponding metabolites in each group under study. A1: treatment with 1000 mg·L^−1^ ABA; A2: treatment with 600 mg·L^−1^ ABA; A3: treatment with 200 mg·L^−1^ ABA; J1: treatment with 800 µmol·L^−1^ MeJA; J2: treatment with 200 µmol·L^−1^ MeJA; J3: treatment with 50 µmol·L^−1^ MeJA; C: control. Values are the mean of three replicates.

Volatile compounds play a key role in wine grapes and wine. In this research, 33 volatile compounds were detected in grape berries. The total volatile content, including alcohols (12), aldehydes (8), esters (5), and ketones (5), was higher in the treated samples than in the control group (Table S2). Among the volatile compounds, C6 and C9 aldehydes and alcohols were significantly affected by the exogenous ABA and MeJA treatments (Table S2 and Figure 1). C6 aldehydes and alcohols, including 2-methyl-cyclopentanol, 1-hexanol, 3-hexen-1-ol, (*E*)-2-hexen-1-ol, hexanal, and (*E*,*E*)-2,4-hexadienal, give grape and wine their grassy and green aromas, while C9 aldehydes and alcohols, including 1-nonen-3-ol, 2,6-dimethylbenzaldehyde, formic acid, octyl ester, and isophorone, provide flower-like aromas. C6 and C9 aromas are mainly formed in the LOX pathway (or fatty acid metabolic pathway) [30]. After exogenous ABA treatment, the total C6 aromas content, ranging from 406.47 μg L^−1^ to 474 μg L^−1^, was higher than in the control group (329.99 μg L^−1^) (Table S2), whereas the samples treated at high ABA concentrations presented a C9 aroma content higher than that in the control (mainly isophorone, formic acid, and octyl ester). Compared to ABA, MeJA may play a more important role in the formation of volatile aromas [5]. After treatment with different concentrations of MeJA, the content of C6 aromas was improved significantly. The C6 aroma content increased with the MeJA concentration (Table S2). That may be related to improvement of the LOX activity (Figure 2) [4]. Interestingly, exogenous ABA and MeJA treatments improved the content of 1-hexanol, hexanal, and 2-heptanol, giving grape berries more varietal aromas (Figure 3). According to Figure S1, the alcohol content was significantly and positively correlated to the aldehyde content in the skin of the Cabernet Sauvignon berries. The 1-hexanol, 2-methyl-cyclopentanol, and 2-propyl-1-pentanol contents were significantly and positively correlated to the 2-hexenal, (*S*)-2,3-dihydroxy-propanal, formic acid, and heptyl ester contents. The 2-methyl-cyclopentanol, (*E*)-2-hexenal, and 2-propyl-1-pentanol content was positively correlated to that of most esters and aldehydes. We found that 2-heptanol and 2,6-dimethylbenzaldehyde were positively correlated with each other, but negatively correlated with other aromas. Furthermore, there were also negative relationships between esters and ketones; in particular, isophorone was significantly and negatively correlated to the formic acid, heptyl ester, and 2-propenylidene-cyclobutene contents. 2-Octanone was negatively correlated to 4-methyl-2-hexanone, propanoic acid, ethyl ester, and *cis*-1,3-cyclohexanediol. 

### 2.5. Multivariate Statistical Analysis of Anthocyanins and Volatile Aromas

**Figure 4:**
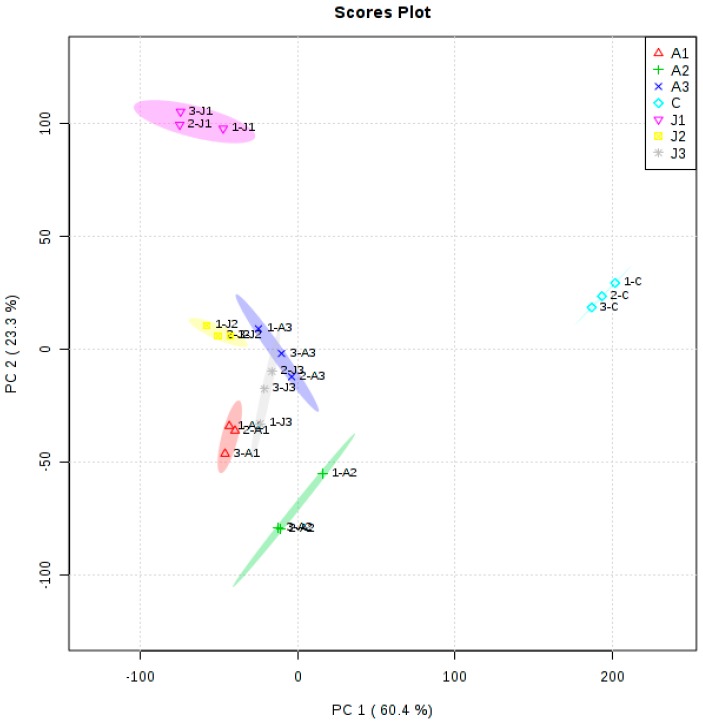
PCA scores plot of samples after different treatments. The colored dots represent samples from different treatments. Seven independent treatment studies, each with triplicate samples, were used in the analysis. A1: treatment with 1000 mg·L^−1^ ABA; A2: treatment with 600 mg·L^−1^ ABA; A3: treatment with 200 mg·L^−1^ ABA; J1: treatment with 800 µmol·L^−1^ MeJA; J2: treatment with 200 µmol·L^−1^ MeJA; J3: treatment with 50 µmol·L^−1^ MeJA; C: control.

Cluster analysis allowed the evaluation of the samples collected after different treatments. The important metabolites affecting the cluster formation in these samples could be determined. Multivariate statistical analysis PCA was applied. The first two principal components (PC1 and PC2) explained about 83.7% of the total variance. Anthocyanins and volatiles influenced the cluster formation. These compounds included anthocyanins, alcohols, aldehydes, and esters (Table S2 and Figure 4). A different cluster pattern from that of the metabolites was observed with the PCA analysis. The control group data were separated from the results of the other six treatments (Figure 4). The A3, J2, and J3 treatments clustered together and were clearly separated from other profiles (Figure 4). The J1 and A2 treatments were also clearly separated from the other treatments, possibly because J1 and A2 treatments change the anthocyanin and volatile aroma content.

## 3. Materials and Methods

### 3.1. Field Conditions and Materials

Six-year-old Cabernet Sauvignon (*Vitis vinifera* L.) vines grafted on SO4 of similar vigor were used in this study. The study was carried out in 2015 in a commercial vineyard located in the territory of Yinchuan (NingXia, China, 38°34′ N 106°1′ E). Vines were spaced at 3.8 m × 1.5 m and drip irrigated. A randomized block design was carried out with three blocks and seven treatments, and each treatment in the block consisted of fifteen individual vines. Following our preliminaries studies, three ABA concentrations (200, 600, and 1000 mg·L^−1^) and three MeJA concentrations (50, 200, and 800 µmol·L^−1^) were used. Pure water treatment was used as the control. On a serene evening with calm wind, the ABA and MeJA treatments were applied to the surface of grape berries on consistently and moderately growing grape vines during the early stages of grape veraison.

Grape samples were collected randomly on September 20th when the berry Brix reached values of 21–24 °Brix. Three hundred berries of each treatment were collected and considered as one replicate, and every collection had three biological replicates. All samples were frozen in liquid nitrogen immediately and stored at −80 °C before further treat.

### 3.2. Determination of Brix, pH, Total Phenolic (TPC), and Total Anthocyanin Content (TAC)

Brix values were determined with a hand-held digital Atago PAL-1 meter (Atago Co. Ltd., Tokyo, Japan) and the pH was measured with a Mettler Toledo FE20 Desktop pH Meter (Mettler Toledo Instruments Co. Ltd., Shanghai, China) [31]. The TPC was determined by the method by Xu et al. and the TAC was estimated according to the pH differential method by Meng et al. [32,33].

### 3.3. Determination of Anthocyanins

#### 3.3.1. Extraction of Anthocyanins

Fifty berries per replicate were selected for each treatment to extract anthocyanins. The grapes were frozen in liquid nitrogen and the skins were removed from the pulp. Then, the berry skins were freeze-dried at −50 °C.

Anthocyanins were extracted according to the methods by Cheng et al. [20] and Zoratti et al. [34]. Grape skin powder (0.50 g) was extracted with 10 mL of a 1 mol·L^−1^ HCl/methanol/water (1:80:19, *v*/*v*/*v*) mixture. The extraction was performed under ultrasound for 10 min at 100 Hz, following by shaking in the dark at 25 °C for 30 min at a rate of 150 rpm. Then, the sample was centrifuged using a high-speed refrigerated centrifuge for 10 min at 10,000 rpm and 4 °C and the supernatant was collected. The residues were re-extracted four times. All the supernatants were collected and combined, concentrated to dryness using a rotary evaporator, and then redissolved in 10 mL with mobile phase A (see below). Before HPLC analysis, all extracts were passed through 0.45 μm filters (Micro Pes, Membrana, Wuppertal, Germany). Each sample was subjected to three independent extractions from three biological replicates.

#### 3.3.2. HPLC Analysis of Anthocyanins

HPLC analysis was performed on a Surveyor HPLC system with a PDA detector (Thermo Finnigan, San Jose, CA, USA). The column was Gemini C18 (250 mm × 4.6 mm; Phenomenex, Torrance, CA, USA) operated at 30 °C. Phase A: water/acetonitrile/formic acid (80:10:7 *v*/*v*/*v*); phase B: water/acetonitrile/formic acid (40:50:7 *v*/*v*/*v*). Samples were eluted according to the gradient described by Wang et al. [35] and Veberic et al. [36] with some modifications. Briefly: 0 min, 0% B; 15 min, 30% B; 25 min, 50% B; 35 min, 0% B, with an injection amount of 10 μL and a flow rate of 1 mL·min^−1^. The anthocyanins were identified by comparing their UV-VIS spectra from 200 to 600 nm to known standards and their retention times; they were detected and quantified at 535 nm.

### 3.4. Determination of the LOX Activity

#### 3.4.1. Extraction of the Crude Enzyme

An appropriate amount of preserved grape skin was immediately ground in liquid nitrogen. Next, 2 g of the powdered grape skin was placed in a centrifuge tube. After adding 4 mL of extraction phosphate buffer (pH 6.8), the mixture was centrifuged using a high-speed refrigerated centrifuge for 30 min at 4 °C and 12,000 rpm. The resulting supernatant was collected to determine the lipoxygenase enzyme activity.

#### 3.4.2. Determination of the LOX Enzyme Activity

The LOX activity was evaluated using the method by Ju et al. [37]. A reaction mixture of 33 μL of substrate (0.5% (*v*/*v*) sodium linoleate solution) in 900 μL of 0.1 mol·L^−1^ phosphate buffer (pH 6.8) was heated at 30 °C in a water bath for 10 min before adding 67 μL of the crude enzyme extract. The absorbance of the mixture was immediately recorded at 234 nm after 30, 45, 60, 75, 90, 105, 120, 135, and 150 s. The enzyme activity has been expressed as △OD_234_ g^−1^ FW min^−1^, and each sample measurement was replicated three times. The UV spectrophotometer was warmed up and set to zero using distilled water before the analysis.

#### 3.4.3. Calculation of the LOX Enzyme Activity

The absorbance values at 234 nm at different times during the reaction were recorded to produce an OD_234_ time series. According to the initial linear portion of the curve from I (starting point) to F (end point), the change in absorbance per minute (△OD_234_) was calculated:

△OD_234_ = (OD_234F_ − OD_234I_)/(*T_F_* − *T_I_*) (1)

Next, one unit of lipoxygenase activity was defined as a 0.01 increment of absorbance in the fruit and vegetable samples (fresh weight) per gram per minute.

*U* = (△OD_234_ × *V*)/(0.01 × *V*s × *m*) (2)

*V*—total sample extraction volume, mL

*V*s—sample extraction volume in the determination, mL

*m*—sample mass, g

### 3.5. Determination of Fatty Acids

#### 3.5.1. Fatty Acid Extraction

The extraction method was modified according to Curtis [38]. First, 5 g of grape skin was immediately and finely ground in liquid nitrogen. Then, a 10 mL mixture of petroleum ether and diethyl ether (4:3, *v*/*v*) was added at 4 °C for 24 h before the extraction. Next, 10 mL of a 0.4 mol·L^−1^ potassium hydroxide/methanol solution was added, methyl etherification was performed for 2 h at room temperature, and the resulting sample was centrifuged at room temperature (25 °C) at 4000 rpm for 10 min. After centrifugation, the organic phase in the upper layer was placed in a 10 mL distillation flask, distilled under reduced pressure, and diluted with distilled water to 5 mL. The experimental setup comprised 1 mL of the sample solution and 1 μL of 100 mg·mL^−1^ methyl heptadecanoate as the internal standard.

#### 3.5.2. GC-MS Analysis of the Fatty Acid Composition

The fatty acid composition was determined by thermoelectric TRACE DSQ gas chromatography-mass spectrometry (GC-MS) according to the method by Ju et al. [39] and Chen et al. [30]. Briefly, the extraction solution was subjected to gas chromatography (Thermo/Finnigan Trace GC Ultra, Thermo Finnigan, Bremen, Germany) on a DB-WAX column (0.25 mm I.D., 30 m, 0.25 μm; Agilent). Nitrogen was used as the carrier gas in the gas chromatographer equipped with a flame ionization detector (FID). The gas velocities were 1 mL·min^−1^ for N_2_, 35 mL·min^−1^ for H_2_, and 350 mL·min^−1^ for air. The GC conditions consisted of an initial temperature of 180 °C for 2.0 min, a final temperature of 240 °C at a rate of 8 °C·min^−1^, an injection temperature of 200 °C, an injection volume of 1 μL, and a split ratio of 80:1.

### 3.6. Determination of Volatile Compounds

#### 3.6.1. Volatile Compound Extraction

Volatile compound extraction and analysis were carried out as described in the literature [40]. Briefly, 100 berries without seeds were grounded and blended with 1 g PVPP in liquid nitrogen. After that, samples were macerated for 2.5 h at 4 °C and then centrifuged at 10,000 rpm at 4 °C for 10 min to collect the supernatant. Then, 1 g NaCl was added to 5 mL of clear juice with 20 μL of the internal standard 2-octanol (0.32 g·L^−1^ in ethanol) and blended in a 15 mL sample vial tightly capped with a PTFE-silicon septum containing a magnetic stirrer for further determination.

#### 3.6.2. GC-MS Analysis of Volatile Compounds

For solid-phase micro extraction (SPME), the extracted fiber was heated at 250 °C for 2 h. The volatile compounds were extracted in a 40 °C water bath for 30 min and subsequently desorbed at 230 °C for 3 min into the splitless injection port of a GC-MS instrument (Thermo-Finnigan Trace 2000/Polaris Q GC/MS, Thermo Finnigan, Shanghai, China) fitted with an HP-INNW AX column (0.25 mm I.D., 60 m, 0.25 μm; Agilent, Shanghai, China). The chromatographic conditions consisted of an initial oven temperature of 40 °C for 2.5 min, a final oven temperature of 230 °C for 7 min, and a temperature increase rate of 6 °C·min^−1^. Nitrogen was used as the carrier gas at 1 cm·s^−1^.

The volatile compounds were identified using the NIST 2002 mass spectrum library (National Institute of Standards and Technology, Gaithersburg, MD, USA), linear retention indices, data in the literature, and mass spectra of pure standards whenever possible. For quantification, characteristic quantifier ions were selected for each compound [31]. All samples were analyzed in triplicate.

### 3.7. Statistical Analysis

All determinations were carried out in triplicate and the results are expressed as mean ± standard deviation (SD). The SPSS 17.0 software (SPSS Inc., Chicago, IL, USA) was used to compare the difference between means by Duncan’s *t*-tests. Values of *p* < 0.05 were considered as significant. Heat maps, principal component analysis (PCA), and partial least squares-discriminant analysis (PLS-DA) were performed with the R pls package.

## 4. Conclusions

In conclusion, after exogenous ABA and MeJA treatments, grape berries exhibited higher TAC and TPC. In terms of individual anthocyanins, the contents of delphinidin-3-*O*-glucoside, malvidin-3-*O*-glucoside, peonidin-3-*O*-glucoside, and cyanidin-3-*O*-glucoside were significantly increased after treatment, in particular after the ABA treatment. Previous research showed that exogenous hormones might regulate the expression of genes involved in many metabolic pathway. Thus, we hypothesize that exogenous ABA and MeJA treatments regulate the expression of genes involved in anthocyanin synthesis, and so affect the content of anthocyanins in grape berries. In particular, exogenous ABA and MeJA improved the LOX activity, thus catalyzed more the conversion of linoleic acid into volatile aromas. Interestingly, the contents of C6 aldehydes and alcohols (in particular, 1-hexanol, hexanal, and 2-heptanol) increased significantly after exogenous ABA and MeJA treatment. Other research found that LOX involved in fatty acid metabolic pathway and catalyzed fatty acid into small molecules, while our results demonstrated that exogenous ABA and MeJA affected the LOX pathway, improving the production of volatile aromas derived from fatty acids. In summary, exogenous ABA and MeJA provide Cabernet Sauvignon grape berries with better varietal aroma and quality in some important aspects of wine production, such as berry characteristics, anthocyanin profiles, and volatile aromas. However, further research on the mechanism of the effect of exogenous ABA and MeJA on the synthesis of anthocyanins and volatile aromas formed in the LOX pathway in grape berries is still necessary.

## Figures and Tables

**Table 1:** Physico-chemical properties of Cabernet Sauvignon berries with different treatments.

Variable	ABA			MeJA			C
	A1	A2	A3	J1	J2	J3	
Brix	21.83 ± 0.12 a	21.97 ± 0.12 a	22.17 ± 0.06 a	21.03 ± 0.40 a	22.03 ± 0.06 b	21.23 ± 0.08 a	21.13 ± 0.29 a
pH	3.95 ± 0.03 a	4.01 ± 0.01 a	3.92 ± 0.01 a	3.82 ± 0.02 b	3.94 ± 0.01 a	3.91 ± 0.03 a	3.97 ± 0.01 a
Total Acid (g L^−1^)	3.65 ± 0.03 a	3.57 ± 0.06 a	3.47 ± 0.04 b	3.66 ± 0.05 a	3.67 ± 0.30 a	4.07 ± 0.06 c	3.49 ± 0.01 b
TPC (mg·g^−1^)	21.18 ± 0.28 b	20.29 ± 0.24 c	19.18 ± 0.06 d	22.19 ± 0.05 b	21.93 ± 0.10 c	19.18 ± 0.06 c	18.19 ± 0.28 a
TAC (mg·g^−1^)	12.28 ± 0.34 b	10.53 ± 0.08 c	9.81 ± 0.32 d	9.82 ± 0.10 b	9.56 ± 0.03 b	9.60 ± 0.15 b	8.85 ± 0.09 a

Note: Values are the mean of three replicates (±standard deviation). A1: treatment with 1000 mg·L^−1^ ABA; A2: treatment with 600 mg·L^−1^ ABA; A3: treatment with 200 mg·L^−1^ ABA; J1: treatment with 800 µmol·L^−1^ MeJA; J2: treatment with 200 µmol·L^−1^ MeJA; J3: treatment with 50 µmol·L^−1^ MeJA; C: control. Different letters within a column indicate statistically significant differences between the means (*p* < 0.05).

**Table 2:** Individual anthocyanin content of Cabernet Sauvignon berry skins after different treatments (mean ± SD, *n* = 3, mg·L^−1^).

Treatments	Delphinidin-3-*O*-glucoside	Cyanidin-3-*O*-glucoside	Petunidin-3-*O*-glucoside	Peonidin-3-*O*-glucoside	Malvidin-3-*O*-glucoside	Non-Acylated (%)	Peonidin-3-*O*-(6-*O*-acetyl)-glucoside	Malvidin-3-*O*-(6-*O*-acetyl)-glucoside	Acetyl Derivatives (%)	Peonidin-3-*O*-(6-*O*-coumaryl)-glucoside	Malvidin-3-*O*-(6-*O*-coumaryl)-glucoside	Coumaryl Derivatives (%)
C	171.68 ± 3.91 a	40.32 ± 0.78 a	100.47 ± 2.35 a	107.88 ± 2.45 a	565.77 ± 12.98 a	72.29	30.90 ± 0.59 a	266.09 ± 6.75 ab	21.77	13.29 ± 0.67 a	67.70 ± 3.34 a	5.94
J1	130.17 ± 3.19 b	23.70 ± 0.50 b	88.63 ± 2.54 b	110.32 ± 3.35 a	678.66 ± 19.14 b	68.50	30.44 ± 1.05 a	334.56 ± 12.19 d	24.24	13.32 ± 0.33 a	96.03 ± 3.25 d	7.26
J2	177.82 ± 5.55 a	38.06 ± 0.66 a	107.52 ± 3.85 ac	109.53 ± 3.39 a	617.42 ± 26.11 a	71.16	30.71 ± 1.16 a	298.41 ± 14.08 c	22.30	14.22 ± 0.83 a	82.27 ± 4.98 c	6.54
J3	189.55 ± 9.34 a	49.12 ± 5.89 c	112.94 ± 5.61 cd	146.90 ± 18.30 b	617.04 ± 26.96 a	72.64	40.89 ± 5.17 c	288.86 ± 10.60 bc	21.47	15.80 ± 1.60 a	74.56 ± 2.76 abc	5.88
A1	217.68 ± 3.20 c	61.94 ± 0.96 d	123.55 ± 2.24 d	138.56 ± 2.46 b	589.32 ± 12.32 a	73.50	37.94 ± 0.64 c	284.51 ± 6.05 bc	20.95	14.54 ± 0.24 a	70.88 ± 1.64 ab	5.55
A2	216.52 ± 7.66 c	62.60 ± 1.85 d	123.03 ± 3.86 d	158.81 ± 4.43 b	597.93 ± 15.09 a	75.48	37.77 ± 0.97 b	248.15 ± 6.14 a	18.62	18.74 ± 0.48 b	71.82 ± 1.45 ab	5.90
A3	176.41 ± 14.54 a	33.93 ± 7.04 a	107.05 ± 8.31 ac	105.55 ± 19.10 a	608.18 ± 37.50 a	71.16	30.22 ± 5.16 a	296.49 ± 14.58 c	22.55	13.44 ± 1.78 a	77.76 ± 3.91 bc	6.29

Notes: A1: treatment with 1000 mg·L^−1^ ABA; A2: treatment with 600 mg·L^−1^ ABA; A3: treatment with 200 mg·L^−1^ ABA; J1: treatment with 800 µmol·L^−1^ MeJA; J2: treatment with 200 µmol·L^−1^ MeJA; J3: treatment with 50 µmol·L^−1^ MeJA; C: control. Different letters within a column indicate statistically significant differences between the means (*p* < 0.05).

**Table 3:** Fatty acid composition in Cabernet Sauvignon berries after different treatments (mean ± SD, *n* = 3, mg·Kg^−1^).

Fatty acid	C	A1	A2	A3	J1	J2	J3
linoleic acid	138.23 ± 7.92 a	116.75 ± 8.40 b	178.08 ± 5.30 c	147.3 ± 9.23 d	127.32 ± 8.35 e	136.72 ± 3.26 a	120.49 ± 5.24 b
elaidic acid	72.22 ± 7.22 a	90.02 ± 5.37 b	102.75 ± 3.42 c	124.70 ± 6.29 d	109.12 ± 7.39 cd	104.33 ± 0.28 c	104.44 ± 6.27 c
9-hexadecenoic acid	0.61 ± 0.052 a	1.16 ± 0.058 b	1.15 ± 0.082 b	0.89 ± 0.032 c	1.09 ± 0.069 d	0.89 ± 0.072 c	1.05 ± 0.127 d
hexadecanoic acid	126.67 ± 9.03 a	146.72 ± 7.93 b	187.36 ± 8.19 c	164.58 ± 5.09 d	159.26 ± 5.83 e	147.43 ± 6.83 b	193.17 ± 5.23 ce
stearic acid	15.34 ± 1.14 a	20.02 ± 1.62 b	21.00 ± 1.78 bc	21.88 ± 1.29 bc	21.02 ± 1.42 bc	15.84 ± 1.52 a	28.21 ± 1.37 d
docosanoic acid	7.17 ± 0.51 a	11.99 ± 0.38 b	10.99 ± 0.69 bc	10.66 ± 0.59 bc	13.27 ± 0.61 d	7.53 ± 0.26 a	15.89 ± 0.39 d
eicosanoic acid	3.40 ± 0.21 a	5.07 ± 0.26 b	4.55 ± 0.19 c	5.42 ± 0.32 de	5.29 ± 0.33 e	4.21 ± 0.31 c	3.18 ± 0.19 a
PUFA	211.06 ± 8.92 a	207.93 ± 7.98 a	281.98 ± 10.61 b	272.89 ± 9.45 c	237.53 ± 6.98 d	241.94 ± 7.46 d	225.98 ± 7.34 e
MUFA	152.58 ± 7.23 a	183.8 ± 8.79 b	223.9 ± 7.84 c	202.54 ± 10.54 bc	198.84 ± 9.57 c	175.01 ± 7.98 d	240.45 ± 7.21 e

Notes: A1: treatment with 1000 mg·L^−1^ ABA; A2: treatment with 600 mg·L^−1^ ABA; A3: treatment with 200 mg·L^−1^ ABA; J1: treatment with 800 µmol·L^−1^ MeJA; J2: treatment with 200 µmol·L^−1^ MeJA; J3: treatment with 50 µmol·L^−1^ MeJA; C, Control. PUFA, polyunsaturated fatty acid; MUFA, monounsaturated fatty acid. Different letters within a column indicate statistically significant differences between the means (*p* < 0.05).

## Supplementary Materials

Supplementary materials can be accessed at: http://www.mdpi.com/1420-3049/21/10/1354/s1.
