# Supplementary material for: Effect of Exogenous Abscisic Acid and Methyl Jasmonate on Anthocyanin Composition, Fatty Acids, and Volatile Compounds of Cabernet Sauvignon (Vitis vinifera L.) Grape Berries
Source: Molecules. 2016 Oct 12;21(10):1354. doi: 10.3390/molecules21101354 (PMC6273220; doi:10.3390/molecules21101354)
Supplement: Supplementary file 1 [file molecules-21-01354-s001.pdf]

Supplementary Materials: Effect of Exogenous Abscisic Acid and Methyl Jasmonate on Anthocyanin Composition, Fatty Acids, and Volatile Compounds of Cabernet Sauvignon (*V. Vinifera* L.) Grape Berries

Yan-Lun Ju, Min Liu, Hui Zhao, Jiang-Fei Meng and Yu-Lin Fang

Table S1. Composition and retention time of berry skin fatty acids (methyl esters).

Name of Fatty Acid	Molecular Formula	Retention Time (min)
tetradecanoic acid	$C_{14}H_{28}O_2$	12.67
pentadecanoic acid	$C_{15}H_{30}O_2$	13.99
9-hexadecenoic acid	$C_{16}H_{30}O_2$	15.08
hexadecanoic acid	$C_{16}H_{32}O_2$	15.42
cyclopropaneoctanoic acid	$C_{17}H_{32}O_2$	16.54
heptadecanoic acid	$C_{17}H_{34}O_2$	16.98
linoleic acid	$C_{18}H_{32}O_2$	18.17
9-octadecenoic acid (elaidic acid)	$C_{18}H_{30}O_2$	18.3
octadecanoic acid (stearic acid)	$C_{18}H_{36}O_2$	18.71
14-methyl octadecanoic acid	$C_{19}H_{38}O_2$	20.63
11-eicosenoic acid	$C_{20}H_{38}O_2$	22.15
eicosanoic acid	$C_{20}H_{40}O_2$	22.71
heneicosanoic acid	$C_{21}H_{42}O_2$	24.88
docosanoic acid	$C_{22}H_{44}O_2$	27.1
tricosanoic acid	$C_{23}H_{46}O_2$	29.37
lignoceric acid	$C_{24}H_{48}O_2$	31.95

Table S2. Mean and standard deviation ($n = 3$) of the amount of volatile aromas in grape berries after different treatments.

Aroma Category	Compounds	Molecular Formula	Volatile Aroma Content (μg·L^−1^)						
			A1	A2	A3	J1	J2	J3	C
alcohols	2-methyl-cyclopentanol	$C_6H_{12}O$	nd	1.02 ± 0.05 a	1.59 ± 0.07 b	4.71 ± 0.16 c	1.17 ± 0.01 a	0.65 ± 0.04 d	6.69 ± 0.13 e
	cis-1,3-cyclohexanediol	$C_6H_{12}O_2$	nd	0.77 ± 0.03	nd	nd	nd	nd	nd
	2-heptanol	$C_7H_{16}O$	0.24 ± 0.05 a	0.27 ± 0.01 a	0.18 ± 0.02 a	0.23 ± 0.06 a	0.27 ± 0.07 a	27.80 ± 0.57 b	0.24 ± 0.05 a
	1-hexanol	$C_6H_{14}O$	22.41 ± 0.07 a	25.61 ± 0.12 b	24.60 ± 0.08 c	45.61 ± 0.72 d	23.45 ± 0.5 e	nd	39.70 ± 0.14 f
	3-hexen-1-ol	$C6H_{12}O$	1.44 ± 0.45 a	0.80 ± 0.01 b	1.11 ± 0.03 d	1.29 ± 0.01 e	0.69 ± 0.03 b	1.31 ± 0.13 e	0.97 ± 0.07 d
	(*E*)-2-hexen-1-ol	$C_6H_{12}O$	17.12 ± 0.85 a	27.06 ± 0.07 b	29.36 ± 0.37 c	37.15 ± 1.48 d	24.41 ± 0.44 e	32.33 ± 0.61 f	28.17 ± 0.1 b,c
	2-octanol	$C_8H_{18}O$	158.69 ± 1.85 a	159.30 ± 0.99 a	158.95 ± 1.48 a	160.55 ± 0.78 a	160.95 ± 1.34 a	159.10 ±1.27 a	160.50 ± 0.71 a
	1-nonen-3-ol	$C_9H_{18}O$	0.40 ± 0.04 a	0.19 ± 0.01 b	0.23 ± 0.03 b,c	0.27 ± 0.01 b	nd	nd	0.25 ± 0.01 b
	6-methyl-5-hepten-2-ol	$C_8H_{16}O$	1.04 ± 0.07 a	nd	0.33 ± 0.01 b	0.18 ± 0.01 c	0.72 ± 0.02 d	0.81 ± 0.04 e	0.72 ± 0.02 d
	2-propyl-1-pentanol	$C_8H_{18}O$	nd	0.21 ± 0.02 a	0.42 ± 0.04 b	0.72 ± 0.04 c	0.33 ± 0.06 d	nd	0.71 ± 0.03 c
	benzyl alcohol	C_7H_8O	0.52 ± 0.08 a	0.45 ± 0.07 a,b,c	0.35 ± 0.05 d	0.49 ± 0.04 a,b	0.37 ± 0.01 e	0.34 ± 0.05 f	nd
	phenylethyl alcohol	$C_8H_{10}O$	0.47 ± 0.08 a	nd	0.30 ± 0.04 b	0.48 ± 0.02 d	0.39 ± 0.03 a,b,c	0.40 ± 0.03 a,d	0.34 ± 0.02 a,b
total			202.33 ± 4.97 a	215.68 ± 2.30 a,b	217.42 ± 4.42 b	251.68 ± 4.64 c	212.75 ± 3.96 d	222.74 ± 5.17 e	238.3 ± 1.56 f
aldehydes	propanal, (*S*)-2,3-dihydroxy	$C_3H_6O_3$	nd	0.77 ± 0.02 a	nd	1.10 ± 0.11 b	nd	nd	0.61 ± 0.05 c
	hexanal	$C_6H_{12}O$	264.83 ± 1.63 a	188.16 ± 1.21 b	189.17 ± 2.27 b	290.88 ± 1.73 c	248.92 ± 1.16 d	215.15 ±1.21 e	30.56 ± 0.09 f
	2-methyl-4-pentenal	$C_6H_{10}O$	2.81 ± 0.1 a	0.13 ± 0.01 b	nd	nd	nd	nd	0.19 ± 0.01 b
	2-hexenal	$C_6H_{10}O$	159.45 ± 0.2 a	168.15 ± 1.20 b	153.20 ± 0.14 c	248.85 ± 1.49 d	161.77 ± 2.5 a	177.39 ± 1.00 e	202.44 ± 1.93 f
	(*E*)-2-hexenal	$C_6H_{10}O$	nd	0.77 ± 0.02 a	0.12 ± 0.01 b	3.23 ± 0.03 c	1.36 ± 0.05 d	1.38 ± 0.02 d	15.32 ± 0.14 e
	(*E*,*E*)-2,4-hexadienal	C_6H_8O	0.18 ± 0.02 a	0.19 ± 0.01 a,b	0.14 ± 0.02 b	0.27 ± 0.01 c	1.30 ± 0.04 c	nd	nd
	benzaldehyde	C_7H_6O	1.30 ± 0.03 a	1.28 ± 0.04 a	1.02 ± 0.1 c	1.50 ± 0.02 d	0.64 ± 0.04 b	1.33 ± 0.05 a	1.62 ± 0.04 b
	2,6-dimethylbenzaldehyde	$C_9H_{10}O$	nd	0.19 ± 0.01 a	0.12 ± 0.01 b	nd	nd	0.22 ± 0.01 c	nd
total			428.56 ± 3.95 a	359.65 ± 2.56 b	343.78 ± 5.07 c	545.83 ± 6.70 d	413.98 ± 7.58 e	395.46 ± 3.55 f	250.74 ± 4.16 g
esters	propanoic acid, ethyl ester	$C_5H_{10}O_2$	1.03 ± 0.06 a	0.42 ± 0.06 b	0.25 ± 0.01 c	0.73 ± 0.02 d	0.56 ± 0.06 e	0.72 ± 0.06 e	0.54 ± 0.02 b
	n-propyl acetate	$C_5H_{10}O_2$	1.83 ± 0.04 a	0.30 ± 0.03 b	0.69 ± 0.02 c	0.40 ± 0.04 d	0.84 ± 0.07 d	1.00 ± 0.02 e	0.26 ± 0.02 b
	formic acid, heptyl ester	$C_8H_{16}O_2$	nd	0.74 ± 0.02 a	nd	1.02 ± 0.14 b	0.64 ± 0.04 a	nd	0.67 ± 0.03 a
	formic acid, octyl ester	$C_9H_{18}O_2$	0.47 ± 0.03 a	0.32 ± 0.01 b	0.42 ± 0.05 a	nd	0.47 ± 0.07 a	nd	0.43 ± 0.03 a
	propyl-propanedioic acid	$C_6H_{10}O_4$	nd	3.73 ± 0.04	nd	nd	nd	nd	nd
total			3.34 ± 0.12 a	5.51 ± 0.16 b	1.36 ± 0.08 c	2.15 ± 0.20 d	2.50 ± 0.23 e	1.72 ± 0.08 f	1.90 ± 0.11 f
ketones	2-octanone	$C_8H_{16}O$	3.99 ± 0.05 a	2.93 ± 0.05 b	3.81 ± 0.08 c	3.67 ± 0.07 c,d	3.53 ± 0.01 d	3.25 ± 0.01 e	2.91 ± 0.03 b
	4-methyl-2-hexanone	$C_7H_{14}O$	nd	0.14 ± 0.01 a	nd	nd	nd	nd	0.17 ± 0.02 b
	6-methyl-5-hepten-2-ol	$C_8H_{16}O$	0.16 ± 0.01 a	nd	0.19 ± 0.01 b	0.18 ± 0.01 b,c	nd	0.07 ± 0.00 a	0.20 ± 0.02 b
	isophorone	$C_9H_{14}O$	0.24 ± 0.01 a	0.18 ± 0.02 b	nd	0.37 ± 0.01 c	0.17 ± 0.03 b	0.38 ± 0.01 c	0.09 ± 0.01 d
	5-ethyl-2(5H)-furanone	$C_6H_8O_2$	0.32 ± 0.01 a	0.36 ± 0.04 a	0.63 ± 0.07 c	0.95 ± 0.03 d	0.60 ± 0.02 b,c	nd	0.52 ± 0.05 b
total			4.71 ± 0.03 a	3.60 ± 0.12 b	4.62 ± 0.16 a	5.16 ± 0.12 c	4.29 ± 0.05 d	3.71 ± 0.11 b	3.88 ± 0.13 e
others	cyclobutene, 2-propenylidene	C_7H_8	1.98 ± 0.00 a	1.82 ± 0.04 b	1.53 ± 0.07 c	1.54 ± 0.05 c	1.34 ± 0.08 d	0.89 ± 0.03 e	2.35 ± 0.05 f
	2-ethyl-furan	C_6H_8O	0.82 ± 0.00 a	0.45 ± 0.01 b	1.01 ± 0.03 c	1.53 ± 0.06 d	2.32 ± 0.06 e	1.14 ± 0.1 f	2.16 ± 0.2 e
	2-hexenoic acid	$C_6H_{10}O_2$	2.90 ± 0.08 a	0.62 ± 0.04 b	3.66 ± 0.07 c	7.28 ± 0.06 d	4.62 ± 0.33 e	4.81 ± 0.07 e,f	4.53 ± 0.08 e
total			5.70 ± 0.09 a	2.89 ± 0.09 b	6.20 ± 0.17 c	10.35 ± 0.17 d	8.29 ± 0.46 e	6.83 ± 0.20 f	9.03 ± 0.33 g
total C6 volatiles			474.00	417.83	406.47	638.98	473.38	432.91	329.99
total C9 volatiles			1.15	0.92	0.77	0.65	0.66	0.61	0.83

Notes: Values are the mean of three replicates (± standard deviation). A1: treatment with 1000 mg·L^{-1} ABA; A2: treatment with 600 mg·L^{-1} ABA; A3: treatment with 200 mg·L^{-1} ABA; J1: treatment with 800 μmol·L^{-1} MeJA; J2: treatment with 200 μmol·L^{-1} MeJA; J3: treatment with 50 μmol·L^{-1} MeJA; C: control; nd: not detected. Different letters within a column indicate statistically significant differences between the means ($p < 0.05$).

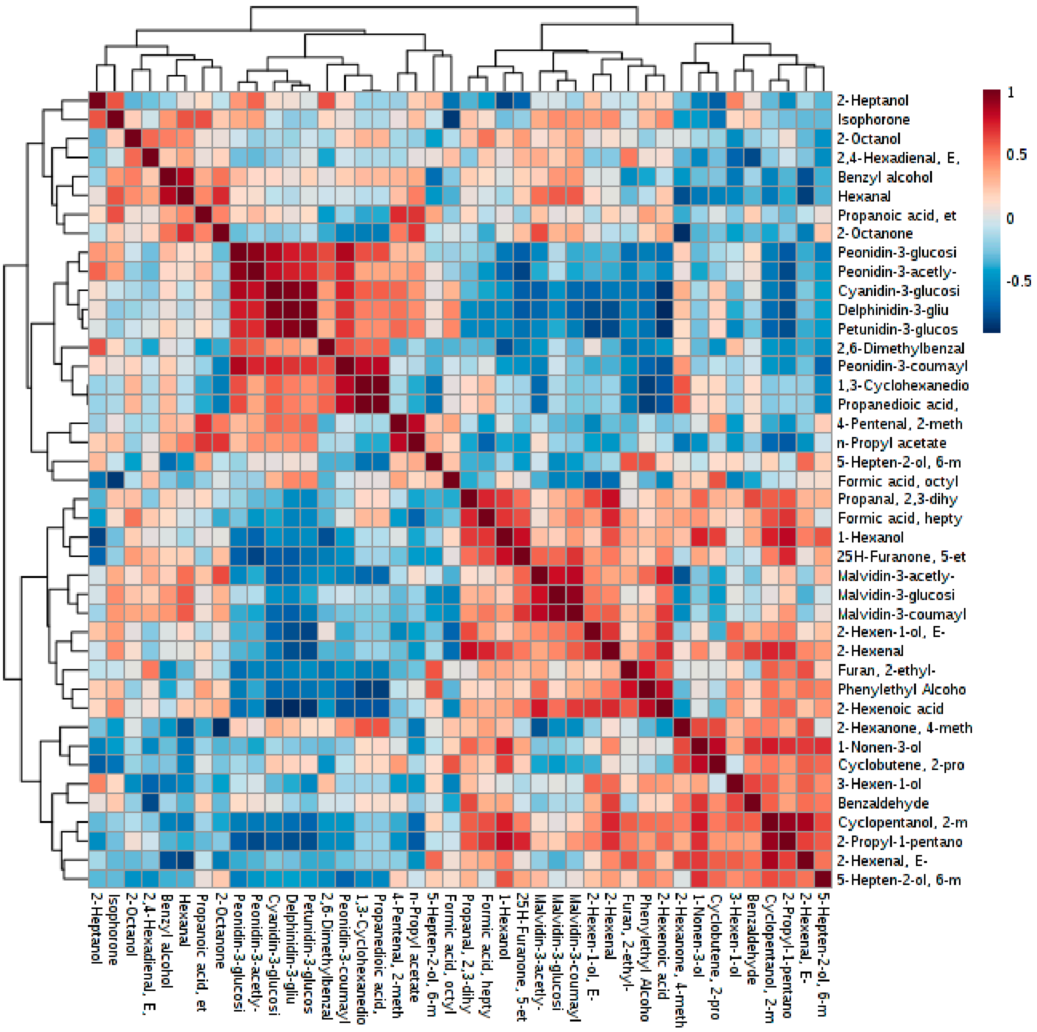

Figure S1. Correlation between different anthocyanins and volatile aromas. The heat map graphic distances were measured using euclidean, and clustering algorithm using ward dendrogram method.
